# Distinct and common expression of receptors for inflammatory mediators in vagal nodose versus jugular capsaicin-sensitive/TRPV1-positive neurons detected by low input RNA sequencing

**DOI:** 10.1371/journal.pone.0185985

**Published:** 2017-10-05

**Authors:** Jingya Wang, Marian Kollarik, Fei Ru, Hui Sun, Benjamin McNeil, Xinzhong Dong, Geoffrey Stephens, Susana Korolevich, Philip Brohawn, Roland Kolbeck, Bradley Undem

**Affiliations:** 1 Department of Respiratory, Inflammatory, and Autoimmunity, MedImmune, LLC, Gaithersburg, Maryland, United States of America; 2 Division of Allergy & Clinical Immunology, Department of Medicine, Johns Hopkins University School of Medicine, Baltimore, Maryland, United States of America; 3 Department of Pathophysiology, Biomedical Center Martin, Jessenius Faculty of Medicine, Comenius University in Bratislava, Martin, Slovakia; 4 Department of Neuroscience, Johns Hopkins School of Medicine, Baltimore, Maryland, United States of America; 5 Department of Translational Medicine, MedImmune, LLC, Gaithersburg, Maryland, United States of America; University of California Los Angeles, UNITED STATES

## Abstract

Capsaicin-sensitive sensory C-fibers derived from vagal ganglia innervate the visceral organs, and respond to inflammatory mediators and noxious stimuli. These neurons play an important role in maintenance of visceral homeostasis, and contribute to the symptoms of visceral inflammatory diseases. Vagal sensory neurons are located in two ganglia, the jugular ganglia (derived from the neural crest), and the nodose ganglia (from the epibranchial placodes). The functional difference, especially in response to immune mediators, between jugular and nodose neurons is not fully understood. In this study, we microscopically isolated murine nodose and jugular capsaicin-sensitive / *Trpv1*-expressing C-fiber neurons and performed transcriptome profiling using ultra-low input RNA sequencing. RNAseq detected genes with significantly differential expression in jugular and nodose neurons, which were mostly involved in neural functions. Transcriptional regulators, including *Cited1*, *Hoxb5* and *Prdm12* showed distinct expression patterns in the two C-fiber neuronal populations. Common and specific expression of immune receptor proteins was characterized in each neuronal type. The expression of immune receptors that have received little or no attention from vagal sensory biologists is highlighted including receptors for certain chemokines (CXCLs), interleukins (IL-4) and interferons (IFNα, IFNγ). Stimulation of immune receptors with their cognate ligands led to activation of the C-fibers in isolated functional assays.

## Introduction

The vagal sensory nervous system plays an important role in maintenance of visceral homeostasis. A large subset of sensory nerves in the vagus nerves can also respond to inflammatory mediators and noxious stimuli [1]. Afferent nerves in the skin that respond to noxious stimuli were coined by Sherrington as “nociceptors” where he concluded they “provide the skin a so to say sense of its own potential injury” [2]. There is an advantage to the host if visceral organs also have a mechanism to provide a sense of its own potential injury, and this may explain why large numbers of visceral afferent nerves have nociceptive properties (are stimulated by inflammatory mediators and noxious stimuli). As with the somatosensory system, visceral nociceptors are typically capsaicin-sensitive slow-conducting C-fibers. Dysregulation of nociceptive function likely participates in the symptoms of visceral inflammatory diseases [3–9]. A better understanding of activating mechanisms of visceral nociceptors may therefore uncover novel targets for new therapeutic strategies for these disorders.

In addition to dorsal root ganglia (DRG), a sizeable proportion of the visceral nociceptive innervation is derived from small capsaicin-sensitive (TRPV1-expressing) C-fiber neurons in vagal sensory ganglia. The majority of vagal C-fibers are derived from neurons situated in the nodose ganglia. Embryologically, these neurons arise from the epibranchial placodes. Another less appreciated component of the vagal C-fiber innervation of visceral tissues is the fibers derived from neurons situated in the jugular (supranodose) ganglia. These neurons arise embryonically not from the epibranchial placodes, but like the spinal afferent neurons in the dorsal root ganglion (DRG), from the neural crest [10]. Whereas a proportion of the jugular neurons are strictly speaking non-vagal as they project their axons to the ear via the auricular nerve, and also to the oral pharynx via pharyngeal nerves, retrograde tracing studies in many mammals including, mice[11, 12], rat[13, 14], guinea pig [15, 16], cats[17, 18], dogs[19] and monkeys[20, 21] reveal numerous jugular neurons that project axons down the vagus nerves. The relative contribution from placodal vs. neural crest vagal afferent innervation likely depends on the visceral organ in question, with thoracic viscera generally receiving a much richer jugular afferent innervation than sub-diaphragmatic viscera [22]. We and others have noted that the respiratory tract, esophagus, and heart are innervated by large numbers of nodose and jugular neurons, most of which have nociceptive properties [22–27]. The nodose and jugular C-fibers share certain C-fiber defining phenotypic properties, including high mechanical threshold and responsiveness to capsaicin. However, the nodose vs. jugular differentiation is important to consider because the receptor expression and activation profile of placodal (nodose) C-fibers differs considerably from neural crest derived (jugular) C-fibers innervating the same tissue [11, 12, 28–32]. Moreover, recent studies are revealing that vagal jugular afferent nerves terminate in distinct regions of the central nervous system from nodose afferent nerves, with the former terminating largely in the paratrigeminal nucleus and the later terminating in the nucleus of the solitary tract [33, 34]. This may explain why activation of jugular and nodose nociceptors within a given tissue, e.g. respiratory tract, can lead to distinctly different reflexes [35].

The nodose and jugular neurons in the mouse typically coexist within an elongated ganglion, which is often inappropriately referred to simply as the nodose ganglion [23]. Although microarrays and RNAseq analyses have been carried out on mouse vagal sensory neurons, these studies likely reflect a mixture of both placodal-derived nodose neurons and neural crest-derived jugular neurons as well as capsaicin-sensitive and–insensitive neurons [36, 37].

In this study we individually isolated capsaicin-sensitive nodose and capsaicin-sensitive jugular vagal sensory neurons using two independent methods, and performed ultra-low input RNA sequencing to characterize the global gene expression in these two types of neurons. To add new insights into this key question we specifically focused our analysis on the expression of receptors for inflammatory mediators.

## Materials and methods

### Isolation of neurons based on capsaicin response

**Fig 1:**
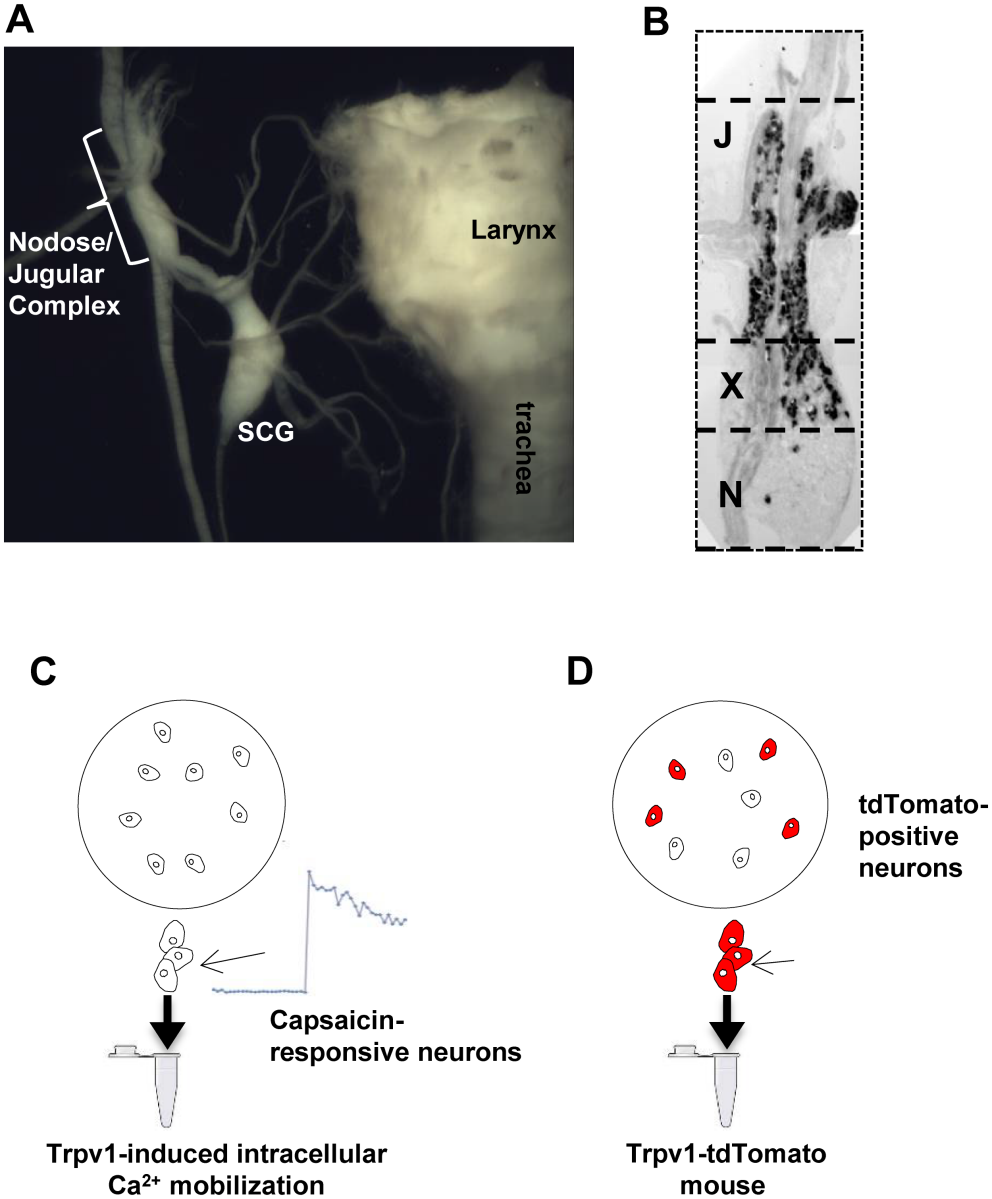
Jugular and nodose C-fiber neurons were isolated based on capsaicin sensitivity and *Trpv1* expression. (A) Dissection of right jugular/nodose complex in the mouse. SCG denotes superior cervical ganglion (modified from [12]. (B) The rostral part of the jugular/nodose ganglion (JNG) complex is formed by jugular (J) neurons while the caudal part is formed by nodose (N) neurons. The jugular (neural crest-derived) neurons are visualized by X-Gal staining while nodose (placodes-derived) neurons remain unstained in Wnt1Cre/R26R mouse [12]. Neurons isolated from the rostral section of the ganglion (J) were considered jugular neurons, whereas those from the more caudal aspect (N) were considered nodose neurons. The upper-central part of the ganglion (X) often comprise a mixture of nodose and jugular neurons and was avoided. (C-D) The collection of capsaicin-responsive / Trpv1-positive C-fiber neurons. The jugular or nodose portions of JNG were enzymatically dissociated, capsaicin-sensitive C-fiber neurons were identified by one of two methods and collected. (C) Identification of C-fiber neurons by capsaicin-responsiveness by intracellular calcium assay in wild-type mice (capsaicin selection). (D) Identification of C-fiber neurons by tdTomato immunofluorescence in the Trpv1-tdTomato mice (Trpv1-tdTomato selection).

All experiments were approved by the Johns Hopkins Animal Use and Care Committee. The right and left vagal sensory ganglia were isolated from wildtype mice. We have previously reported that in mice the jugular and nodose neurons typically form a fused jugular/nodose ganglion (JNG) complex [12] (Fig 1A and 1B). The jugular neurons are situated in the rostral portion of the ganglia, whereas the caudal portion is composed nearly exclusively of nodose neurons (Fig 1B). Therefore, from each JNG the most rostral (jugular) and the most caudal (nodose) portion were used. The ganglia were enzymatically dissociated and the responsiveness of individual neurons to the selective activator of nodose C-fibers, purinergic P2X agonist α,β-methylene-ATP (10 μM) and to the general C-fiber activator TRPV1 agonist capsaicin (1μM) was evaluated by Fura-2 AM intracellular calcium assay as previously described [11, 12]. After determining the responsiveness to agonists, the neurons were collected under the microscope into a glass pipette (pulled to tip diameter 50–150 μm) by applying negative pressure (up to 5 neurons/ pipette). The pipette tip was then broken in a PCR tube containing RNAse inhibitor, immediately frozen on dry ice and stored at -80°C. A total of 31 α,β-methylene-ATP-unresponsive, capsaicin-responsive neurons (jugular C-fiber neurons) from the rostral portion (15 cells were pooled for Replicate1 and 16 cells were pooled for Replicate2) and 29 α,β-methylene-ATP-responsive, capsaicin-responsive neurons (nodose C-fiber neurons) from caudal portion (14 cells were pooled for Replicate1 and 15 cells were pooled for Replicate2) were collected. Care was taken to avoid adhering cells, debris and glial cells.

### Generation of Trpv1-Td-tomato reporter mice and isolation of neurons based on Trpv1 expression

Trpv1-tdTomato mice was generated by mating Trpv1^Cre^ mice with tdTomato reporter strain Gt(ROSA)26Sortm14(CAG-tdTomato)Hze using standard approach. The ganglia were dissected and dissociated the same way as for capsaicin response-based isolation described above. Individual red fluorescent (Td-tomato-positive) neurons from the most caudal nodose portion and from the most rostral jugular portion were collected under fluorescent microscope by using rhodamine (red) filter set. Total of 60 nodose and 60 jugular Td-tomato-positive neurons (30 cells were pooled for each replicate) were collected from 2 mice (Fig 1C). For comparison, 30 Td-tomato-negative nodose neurons were collected from the same mice (15 cells were pooled for each replicate).

### Ultra-low input RNA sequencing

Cells from capsaicin response based isolation (capsaicin-responsive jugular and nodose neurons) were processed at the Johns Hopkins Deep Sequencing and Microarray Core. Cells from TRPV1 expression based isolation (Td-tomato-positive jugular and nodose neurons, and Td-tomato-negative nodose neurons) were processed at MedImmune Deep Sequencing and Microarray Core independently following the same protocol. Briefly, RT-PCR and cDNA synthesis was performed using SMARTer Ultra Low Input RNA Kit v3 following manufacturer’s protocol (Clontech). Cells were used directly as starting material without RNA extraction to avoid extra loss of RNA. First stand cDNA was synthesized using 3’ SMART CDS Primer II A and SMARTer IIA Oligonucleotide with SMARTScribe Reverse Transcriptase. dT priming was used to eliminate DNA contamination. The cDNA was then amplified by 15 cycles of LD PCR and purified using Agencourt AMPure XP kit. Amplified cDNA was validated using Agilent 2100 BioAnalyzer. Illumina paired-end DNA sequencing library was generated using Low Input Library Prep Kit v2 following manufacturer’s protocol (Clontech). The libraries were purified using AMPure beads and quality validated using Agilent 2100 BioAnalyzer. Sequencing was performed on Hiseq 2000. 50bp paired-end sequencing was performed with ~30 million reads generated per sample for capsaicin response based isolation, and ~250 million reads generated per sample for TRPV1 expression based isolation.

### RNA sequencing data analysis

Quality of the RNAseq data, such as the overall sequencing score, over-represented reads, kmer presence, was evaluated using the FastQC package (http://www.bioinformatics.babraham.ac.uk/projects/fastqc/). Sequencing reads were aligned to mouse reference genome mm10 using Tophat2 (v2.0.9) [38] and Bowtie2 (2.1.0.0)[39]. Default parameters were used, allowing maximum of 2 mismatches during alignment. Raw counts were generated using HTseq[40]. Data was normalized and counts per million (CPM) values for each gene were generated using the Deseq2 package. Group comparison was performed in Deseq2 using generalized linear model assuming negative binomial distributions[41]. False discovery rate (FDR) was generated with Benjamini and Hochberg correction. Genes with significant expression change was defined as fold change ≥ 2 and FDR ≤ 0.1. To improve the quality of analysis, additional filtering was applied to only keep the genes with CPM ≥ 10 in both replicates in jugular or nodose or both of the two populations. Functional annotation of the genes was performed in PANTHER classification system (http://pantherdb.org/). Pathway analysis was performed using DAVID (https://david.ncifcrf.gov/tools.jsp) against Gene Ontology biological process and molecular function database. Pathway enrichment FDR was generated from p values with Benjamini and Hochberg correction. Enriched pathways were ranked by FDR of enrichment. Heatmap and Principle component analysis were generated in R using log2 transformed counts per million (CPM) values. Other graphing and statistics were performed using R and Microsoft Office Excel. Raw and processed RNA sequencing data has been uploaded to Gene Expression Omnibus (https://www.ncbi.nlm.nih.gov/geo/) with accession number GSE102123.

### Intracellular Ca2+ measurements

For measurement of intracellular Ca^2+^ concentrations ([Ca^2+^]_i_), nodose neurons were isolated from *Pirt-GCaMP3* heterozygote mice in which the genetically encoded Ca^2+^ indicator GCaMP3 is expressed under the sensory neuron-specific *Pirt* promoter as previously described[42]. Briefly, mice were killed by CO_2_ inhalation and subsequent exsanguinations. Both sides of jugular/nodose ganglia were dissected and cleared of adhering connective tissues. The lower two thirds of ganglia (containing mostly nodose neurons) were cut out for subsequent enzymatic digestion using type 1A collagenase (2mg/ml) and dispase II (2mg/ml). The isolated nodose neurons were kept at 37 C in L-15 medium containing 10% of fetal bovine serum for use within 24 hours. GCaMP3 was excited at 488 nm, and the emission of its green fluorescence at 525 nm was captured by a Nikon DS camera (30–60 frames/min) under the control of imaging software NIS-Elements AR. The images were continuously captured before and during agonist application. The intensity of whole-cell fluorescence for each cell under study was measured as a function of time, normalized to the resting fluorescence (F/F_0_) and used as an index of the [Ca^2+^]_i_. The experiment was performed at room temperature. Bath solution contained (mM): NaCl 136, KCl 5.4, MgCl_2_ 1, CaCl_2_ 1.5, HEPES 10 and glucose 10 with pH adjusted to 7.35 with NaOH.

The stock solution of mouse interferon-α A (PBL Assay Science, Piscataway, NJ), recombinant mouse interference-γ and recombinant human CXCL8 (R&D Systems, Minneapolis, MN) was prepared in PBS containing 0.1% BSA, and sphingosine-1 phosphate (Tocris, Bristol, UK) in ethanol.

### Extracellular recording

The innervated isolated trachea-lung preparation was prepared as previously described [43]. Briefly, the airways and lungs with their vagus nerve including jugular/nodose ganglia were placed in a dissecting dish containing Krebs bicarbonate buffer solution composed of (mM) 118 NaCl, 5.4 KCl, 1.0 NaH_2_PO_4_, 1.2 MgSO_4_, 1.9 CaCl_2_, 25.0 NaHCO_3_ and 11.1 dextrose, and equilibrated with 95% O_2_ and 5% CO_2_ (pH 7.2–7.4). The left jugular/nodose ganglion was gently pulled into the adjacent compartment of the chamber through a small hole and pinned. Both compartments were separately superfused with Krebs solution, which was warmed by a warming jacket (39–42°C) to keep airway tissues and ganglia at 37°C. Action potentials were recorded at the level of the cell body strategically positioned in the vagal sensory ganglion using a sharp extracellular glass electrode filled with 3M NaCl (impedance 2MΩ). The recorded action potentials were amplified (Microelectrode AC amplifier 1800; A-M Systems, Everett, WA, USA), filtered (0.3 kHz of low cut-off and 1 kHz of high cut-off), and monitored on an oscilloscope (TDS340; Tektronix, Beaverton, OR, USA) and a chart recorder (TA240; Gould, Valley View, OH, USA). The scaled output from the amplifier was captured and analyzed by a Macintosh computer using NerveOfIt software (Phocis, Baltimore, MD, USA). We have found that this technique does not record action potentials in “through fibers”. For example, positioning the electrode away from cell bodies on the vagus itself, fails to record action potential stimulated by receptive fields, or be electrical nerve stimulation. For measuring conduction velocity, an electrical stimulation (S44; Grass Instruments, Quincy,MA,USA) was applied on the core of the receptive field. The conduction velocity was calculated by dividing the distance along the nerve pathway by the time delay between the shock artifact and the action potential evoked by electrical stimulation. If a C-fiber (<1 m s^-1^) was found, the recording was started. C-fibers were stimulated by 1 ml of vehicle, α,β-methylene ATP (10 μM), shingosine-1phosphate (S-1P) (0.1, 1 and 10 μM), or Thr-Phe-Leu-Leu-Arg-NH2 (TFLLR) (3 μM) injected into the lung through the trachea.

## Results

### Differential gene expression in Trpv1-tdTomato-positive jugular vs. nodose neurons

We compared the transcriptome between jugular vs. nodose neurons in the capsaicin selection and Trpv1 selection dataset, respectively. Previously, we have studied the function and expression of several ion channels (P2X_2_, P2X_3_, 5-HT_3_)[11, 12, 28, 29], G-protein coupled receptors (PAR1, PAR2, adenosine A_1_ receptor) [31, 32], receptors for neurotrophins and neurotrophic factors (TrkA, TrkB and GFRα_3_) [11, 12] and neuropeptides (PPT-A products) [11, 12] that differentiate between nodose and jugular capsaicin-sensitive/TRPV1-positive C-fiber neurons using functional, immunohistochemical and/or RT-PCR assays. We used this knowledge to evaluate the validity of our approach. The predictions from previous studies were satisfied in every case in the transcriptome analysis of jugular and nodose neurons using either the capsaicin-sensitivity or Trpv1-tdTomato selection to delineate the C-fibers (Table 1 and references therein).

**Table 1:** Concordance between RNAseq analysis^a^ and functional, histological, and single cell RT-PCR analysis^b^.

	Nodose			Jugular		
	Previously established expression^b^	Capsaicin selection^a^(CPM)	Trpv1 selection^a^(CPM)	Previously established expression^b^	Capsaicin selection^a^(CPM)	Trpv1 selection^a^(CPM)
**Ion Channels**						
P2X_3_ (*P2rx3*)	**+**	255	195	**+**	198	214
P2X_2_ (*P2rx2*)	**+**	395	412	**-**	0	0
5-HT_3_ (*Htr3a*)	**+**	223	1135	**-**	20	70
**G protein-coupled receptors**						
PAR1 (*F2r*)	**+**	503	449	**-**	0	14
PAR2 (*F2rl1*)	**-**	0	0	**-**	0	0
A_1_ *(Adora1)*	**+**	78	79	**+**	45	119
**Neurotrophic Factor Receptors**						
TrkA (*Ntrk1*)	+/-	0	30	**+**	60	397
TrkB (*Ntrk2*)	**+**	154	148	+/-	0	16
GFRα_3_ (Gfra3)	**-**	0	0	**+**	130	56
**Neuropeptides**						
PPT-A (*Tac1*)	**-**	0	0	**+**	686	1030

^a^RNAseq was carried in nodose and jugular neurons in two different experimental protocols as detailed in methods. For experiment 1 the capsaicin-sensitivity was determined in a calcium assay. For experiment 2 the capsaicin responsivity was inferred from the capsaicin receptor *Trpv1* expression based on a reporter Trpv1 td-Tomato reporter mouse. CPM: Normalized counts per million values.

^b^The data with the ion channels and GPCRs is based on action potential discharge from identified jugular vs nodose C-fibers terminating in lungs and/or esophagus together with single cell RT-PCR verification; whereas the neurotrophic factor receptors and neuropeptides is based on histology together with single cell RT-PCR verification. The +/- designation reflects where there was only a minority of neurons expressing the neurotrophic factor receptor in single cell RT-PCR analysis. See text for references.

**Fig 2:**
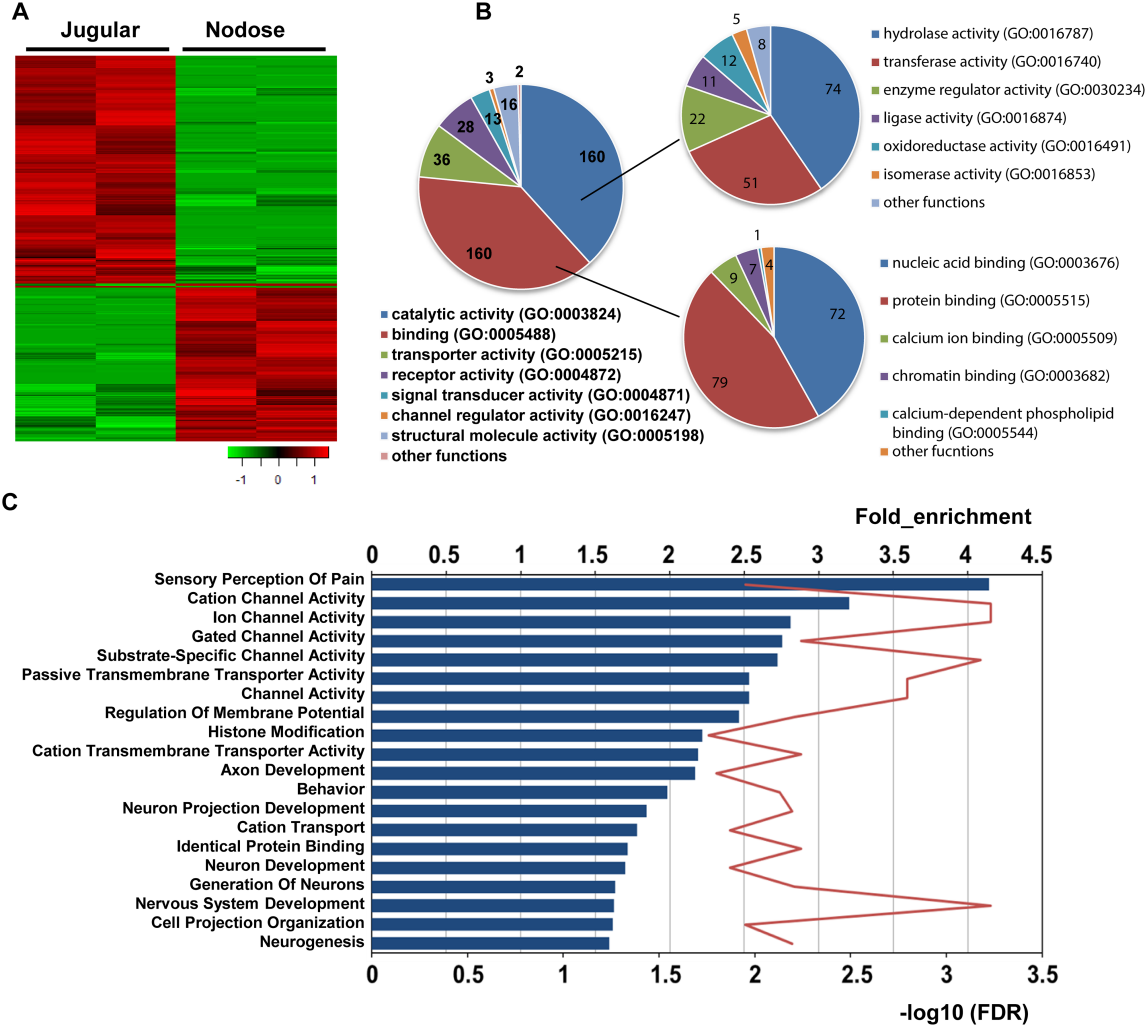
RNA sequencing revealed distinct gene expression between nodose and jugular C-fiber neurons. (A) Heatmap showing expression of genes with significant expression difference between nodose and jugular C-fiber neurons. Log2 transformed CPM was used. Red indicates high expression, green indicates low expression. Color bar indicates z score. (B) Pie plot showing functional annotation of genes with significant expression difference between nodose and jugular C-fiber neurons. The number of genes in each category is indicated. (C) Top 20 Gene Ontology (molecular function and biological process) pathways enriched for genes with significant expression difference between nodose and jugular C-fiber neurons. Pathways are ranked by fold of enrichment. Blue bar and upper horizontal axis indicate fold of enrichment; red line and lower horizontal axis indicate–log10 (FDR). Data from TRPV1 selection dataset.

Group comparison was performed between nodose and jugular TRPV1-positive neurons using the Trpv1 selection dataset because of higher coverage. The analysis revealed 326 genes with significantly higher expression in jugular, and 222 genes with significantly higher expression in nodose TRPV1-positive neurons (fold change ≥ 2 and False discovery rate (FDR) ≤ 0.1) (Fig 2A, S1 Table). Functional annotation of the genes with significant expression difference revealed that the top protein function categories are catalytic activity, binding activity, transporter activity and receptor activity (Fig 2B). Pathway enrichment analysis further revealed that these genes are mostly involved in ion channel activity, membrane transport activity, and neuronal development (Fig 2C).

**Fig 3:**
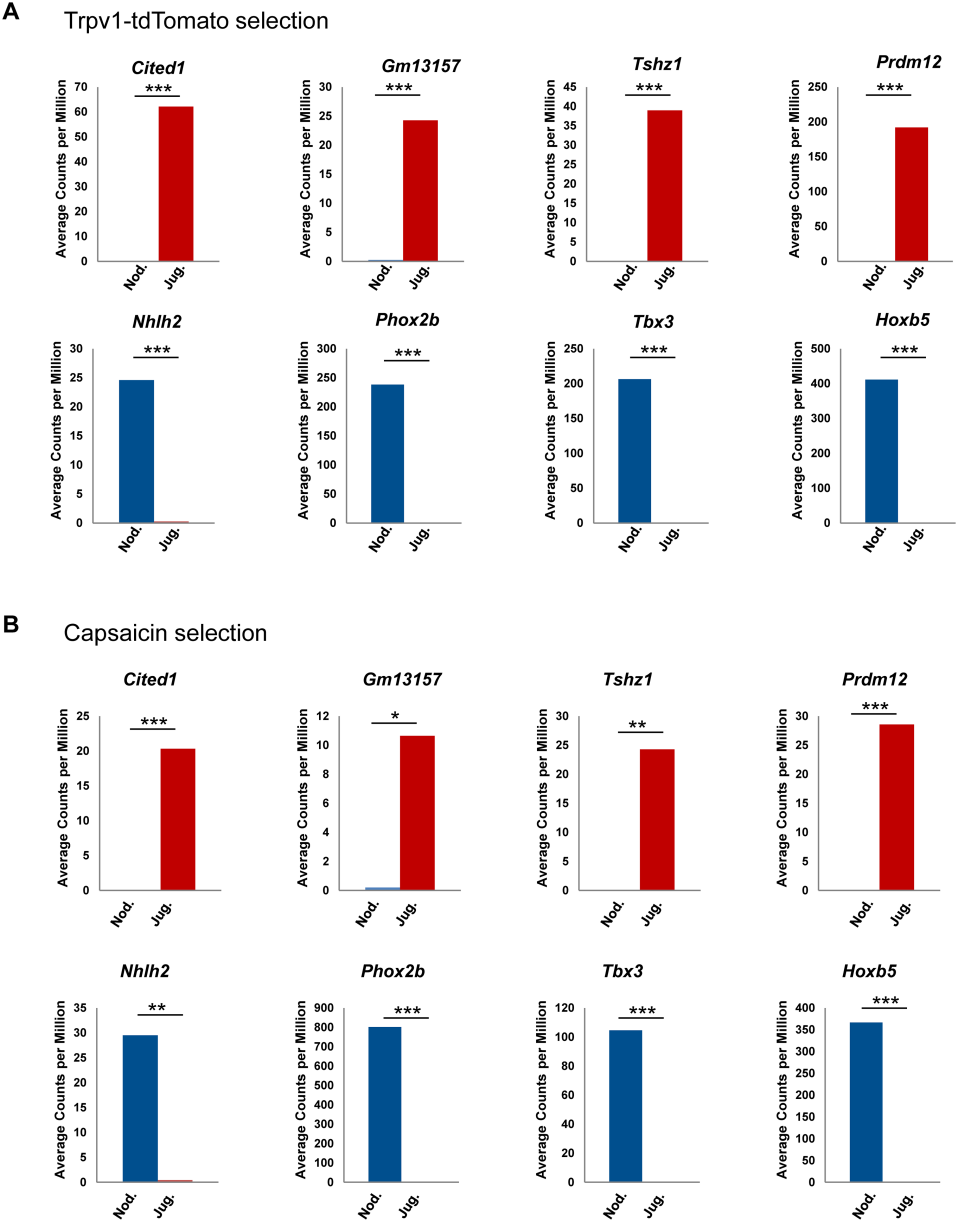
RNA sequencing revealed distinct expression of transcription factors between nodose and jugular C-fiber neurons. (A) Transcription factors differentially expressed in nodose and jugular C-fiber neurons from the Trpv1 selection. Y-axis indicates average of normalized counts per million (CPM) from Trpv1-tdTomato selection. Blue indicates nodose, red indicates jugular C-fiber neurons. P values were calculated based on group comparison. * p value ≤ 0.05; ** p value ≤ 0.01; *** p value ≤ 0.001. (B) Transcription factors differentially expressed in nodose and jugular C-fiber neurons from the capsaicin selection. Y-axis indicates average CPM from capsaicin selection. Blue indicates nodose, red indicates jugular C-fiber neurons. P values were calculated based on group comparison. * p value ≤ 0.05; ** p value ≤ 0.01; *** p value ≤ 0.001.

Notably, RNAseq revealed multiple transcriptional regulators with significant differential expression between nodose and jugular TRPV1-positive neurons (Fig 3A). The transcription regulator *Cited1*, *Gm13157* (predicted murine homolog of *ZNF616* gene in human), *Tshz1* showed predominant expression in jugular, while *Nhlh2*, *Phox2b*, *Tbx3* and *Hoxb5* showed predominant expression in nodose TRPV1-positive neurons. Also, the epigenetic enzyme *Prdm12* was detected in jugular, but not nodose TRPV1-positive neurons. A similar pattern of differential expression of these transcription regulators was also observed in the capsaicin selection dataset (Fig 3B, S2 Table).

We characterized the expression of receptors for inflammatory and immune mediators that may recruit vagal jugular and nodose capsaicin-sensitive C-fiber activity in visceral inflammatory diseases. The receptors for only a small percentage of inflammatory and immune mediators were expressed by jugular or nodose TRPV1-positive neurons; but distinct expression of certain cytokines, chemokines, TRP channels, and lipid mediators were noted in both types of TRPV1-positive neurons (Table 2). Interestingly, there were relatively few receptors for chemokines, cytokines, or toll-like receptors expressed by vagal TRPV1-positive neurons. A notable exception was *Cxcr2* which was found to be expressed in both nodose and jugular TRPV1-positive neurons. Amongst the numerous cytokine receptors, interleukin 4 receptor *Il4ra* was expressed and Type 1 and 2 interferon receptors were strikingly expressed. The sphingosine-1P receptor, *S1pr3*, was the most expressed of all lipid mediator receptors. Among the prostanoids receptors, the prostacyclin receptor *Ptgir* showed stronger expression in nodose neurons, while the prostaglandin D receptor *Ptgdr* was selectively expressed in jugular neurons.

**Table 2:** Expression of receptors for selected immune mediators in nodose and jugular afferent neurons^a^.

Gene	Nodose	Jugular	Gene	Nodose	Jugular	Gene	Nodose	Jugular
**Chemokine receptors**			*Il6ra*	0	0	*Ptgdr2*	0	1
*Cxcr1*	0	0	*Il7r*	0	0	*Ptger1*	4	20
*Cxcr2*	124	163	*Il9r*	0	0	*Ptger2*	11	12
*Cxcr3*	0	0	Other interleukin receptors	0	0	*Ptger3*	0	0
*Cxcr4*	0	0	*Tnfrsf1a*	1	1	*Ptger4*	9	0
*Cxcr5*	1	0	*Tnfrsf1b*	9	0	*Ptgfr*	0	0
*Cxcr6*	0	0	*Ltbr*	30	1	*Ptgir*	86	26
*Cxcr7*	0	0	**Interferon receptors**			*Tbxa2r*	0	0
*Ccr1*	0	0	*Ifnar1*	86	114	**Sphingosine-1P receptors**		
*Ccr2*	0	0	*Ifnar2*	26	5	*S1pr1*	0	3
*Ccr3*	0	0	*Ifngr1*	1	19	*S1pr2*	8	14
*Ccr4*	0	0	*Ifngr2*	119	31	*S1pr3*	376	46
*Ccr5*	0	0	*Il10rb*	48	31	*S1pr4*	3	2
*Ccr6*	0	0	**Toll-like Receptors**			*S1pr5*	0	0
*Ccr7*	0	0	*Tlr1*	0	0	**LPA receptors**		
*Ccr8*	0	0	*Tlr2*	0	0	*Lpar1*	36	61
*Ccr9*	0	0	*Tlr3*	0	9	*Lpar2*	0	0
*Ccr10*	0	0	*Tlr4*	0	0	*Lpar3*	0	51
*Ccr11*	0	0	*Tlr5*	0	0	*Lpar4*	0	0
*Xcr1*	0	0	*Tlr6*	0	0	*Lpar5*	0	0
*Cx3cr1*	14	6	*Tlr7*	0	0	*Lpar6*	0	0
**Cytokine receptors**			*Tlr8*	0	0	**Resolvin Family receptors**		
*Il1r1*	0	0	*Tlr9*	0	0	*Fpr2*	0	0
*Il2ra*	0	0	*Tlr10*	0	0	*Gpr18*	0	0
*Il3ra*	0	1	*Tlr11*	0	0	*Cmklr1*	0	0
*Il4ra*	0	83	**Prostaglandin receptors**					
*Il5ra*	3	2	*Ptgdr*	0	64			

^a^Values in the table indicate mean Counts per Million (CPM) values across replicates.

**Fig 4:**
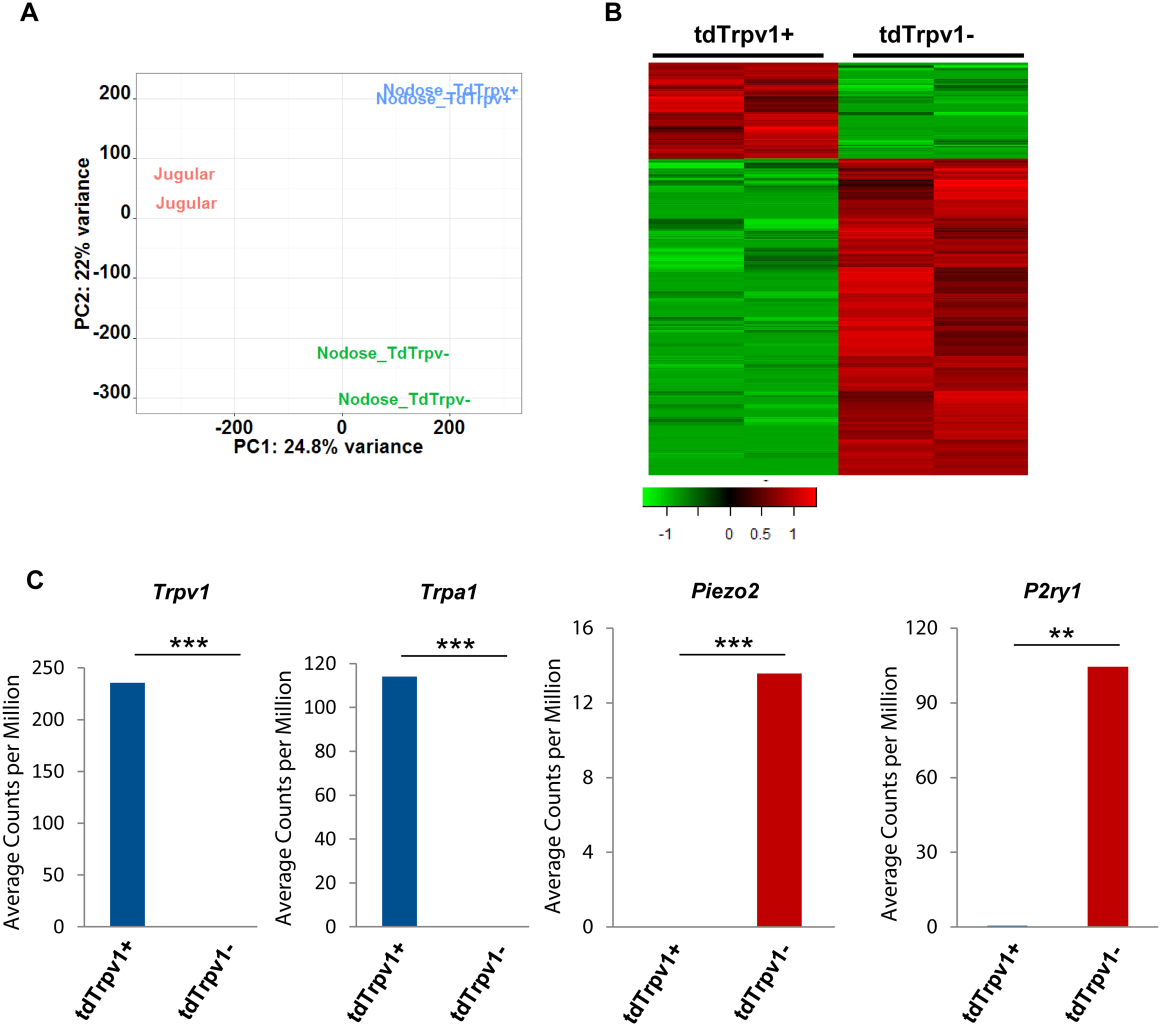
RNA sequencing revealed distinct gene expression between Trpv1-tdTomato positive and negative nodose C-fiber neurons. (A) Principle component analysis showing the overall expression patterns of jugular, Trpv1-tdTomato positive and negative nodose C-fiber neurons. Log2 transformed CPM from Trpv1-tdTomato selection dataset was used. (B) Heatmap showing expression of genes with significant expression difference between Trpv1-tdTomato positive and negative nodose C-fiber neurons. Log2 transformed CPM was used. Red indicates high expression, green indicates low expression. Color bar indicates z score. (C) Selected genes that are differentially expressed in Trpv1-tdTomato positive and negative nodose C-fiber neurons. Y-axis indicates average CPM from Trpv1-tdTomato selection dataset. Blue indicates Trpv1-tdTomato positive nodose neurons, red indicates Trpv1-tdTomato negative nodose neurons. P values were calculated based on group comparison. * p value ≤ 0.05; ** p value ≤ 0.01; *** p value ≤ 0.001. Data from TRPV1 selection dataset.

As additional control we evaluated capsaicin-insensitive neurons. The function of these neurons has not been thoroughly characterized in most visceral organs, however, in the respiratory tract and esophagus these neurons are largely limited to low-threshold mechanosensitive A-fibers that, depending on the nerve phenotype, are sensitive to light touch and/or subtle increases in wall tension [1, 6, 44]. Principle component analysis on the overall transcriptome expression revealed that these capsaicin-insensitive (Trpv1-tdTomato-negative) nodose neurons have distinct gene expression compared to the capsaicin sensitive (Trpv1-tdTomato-positive) nodose and jugular neurons (Fig 4A). Group comparison between Trpv1-tdTomato-negative and -positive nodose neurons detected 99 genes with significantly higher expression in Trpv1-tdTomato-positive nodose C-fiber neurons, and 328 genes with significantly higher expression in Trpv1- tdTomato-negative nodose C-fiber neurons (fold change ≥ 2 and FDR ≤ 0.1) (Fig 4B, S3 Table). As expected, the Trpv1- tdTomato-negative population of nodose neurons also lacks expression of other nociceptor associated TRP channels such as *Trpa1*. Also consistent with putative functions of these neurons, the low-threshold mechanosensitive receptor *Piezo2* [45] is richly expressed in the Trpv1- tdTomato-negative population, but not in the capsaicin-sensitive / Trpv1- tdTomato-positive population of nodose neurons. In addition to *Piezo2* these neurons selectively express *P2ry1*, consistent with the recent findings of Chan and collaborators (Fig 4C) [46].

### Functional consequences of the activation of immune receptors

**Fig 5:**
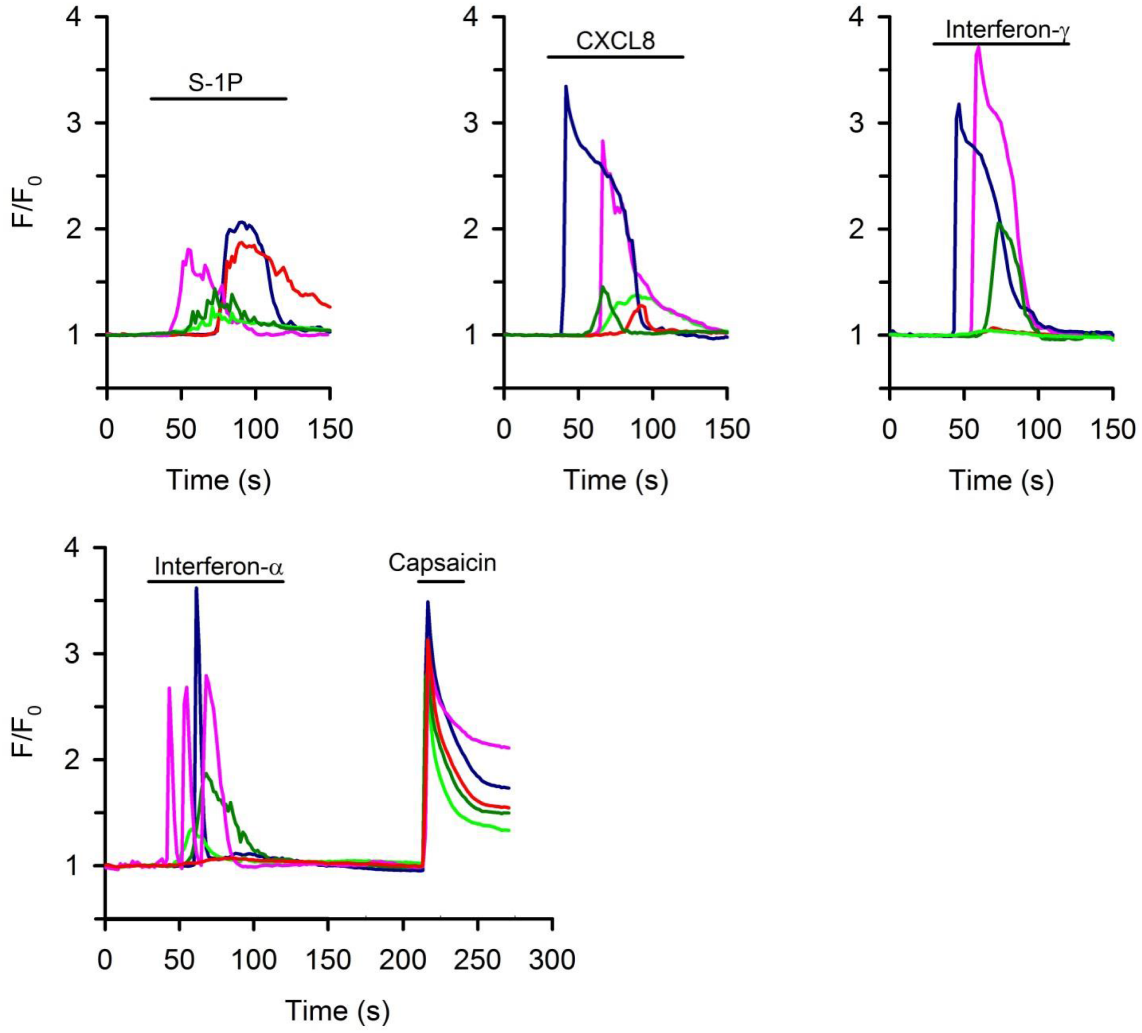
Response of isolated vagal sensory neurons to interferon-α (1000iu/ml) interferon- γ (200 ng/ml) CXCL8 (100ng/ml), and S-1P (10 μM) in a calcium assay. The vagal neurons were isolated from *Pirt-GCaMP3* heterozygote mice in which the genetically encoded Ca^2+^ indicator GCaMP3 is expressed under the sensory neuron-specific *Pirt* promoter. Capsaicin sensitivity was determined at the end of the experiment by adding 1 μM capsaicin. The capsaicin response is shown in the experiments with interferon-α to provide some insight into efficacy of the mediator responses relative to a maximal capsaicin response. Different colors indicate response in different representative neurons (Total of N>100 neurons were tested in each condition). F/F0: The intensity of whole-cell fluorescence for each cell under study as a function of time, normalized to the resting fluorescence.

To determine whether the immune receptors expressed in nodose neurons are functionally important, we have evaluated the effects of their respective agonists on the [Ca^2+^]_i_ given the fact that rapid increases in intracellular Ca^2+^ correlates with nociceptor activation. We evaluated the agonists for those receptors for mediators that have received little attention from sensory biologists. These include interferon-α, interferon-γ, CXCL8 and sphingosine-1 phosphate. Each of these stimuli elicited fast increases in [Ca^2+^]_i_ in 30 to 40% of capsaicin-sensitive neurons for different agonists (Fig 5). Interferon-α, Interferon-γ, CXCL8 and S-1P evoked response in 34.2% (N = 146), 40% (N = 135), 37.6% (N = 117) and 31.5% (N = 143) of capsaicin-sensitive neurons, respectively. The amplitude of increases in the [Ca^2+^]_i_ is variable among different neurons, but in some neurons was as robust as those induced by maximal capsaicin stimulation (Fig 5). In contrast, these stimuli failed to activate capsaicin-insensitive neurons. Only 1 out of 117 and 1 out of 84 neurons that were not capsaicin-sensitive responded to interferon-α and CXCL8, respectively, with a small increase in the Ca^2+^ signal the rest were unaffected by the stimuli.

**Fig 6:**
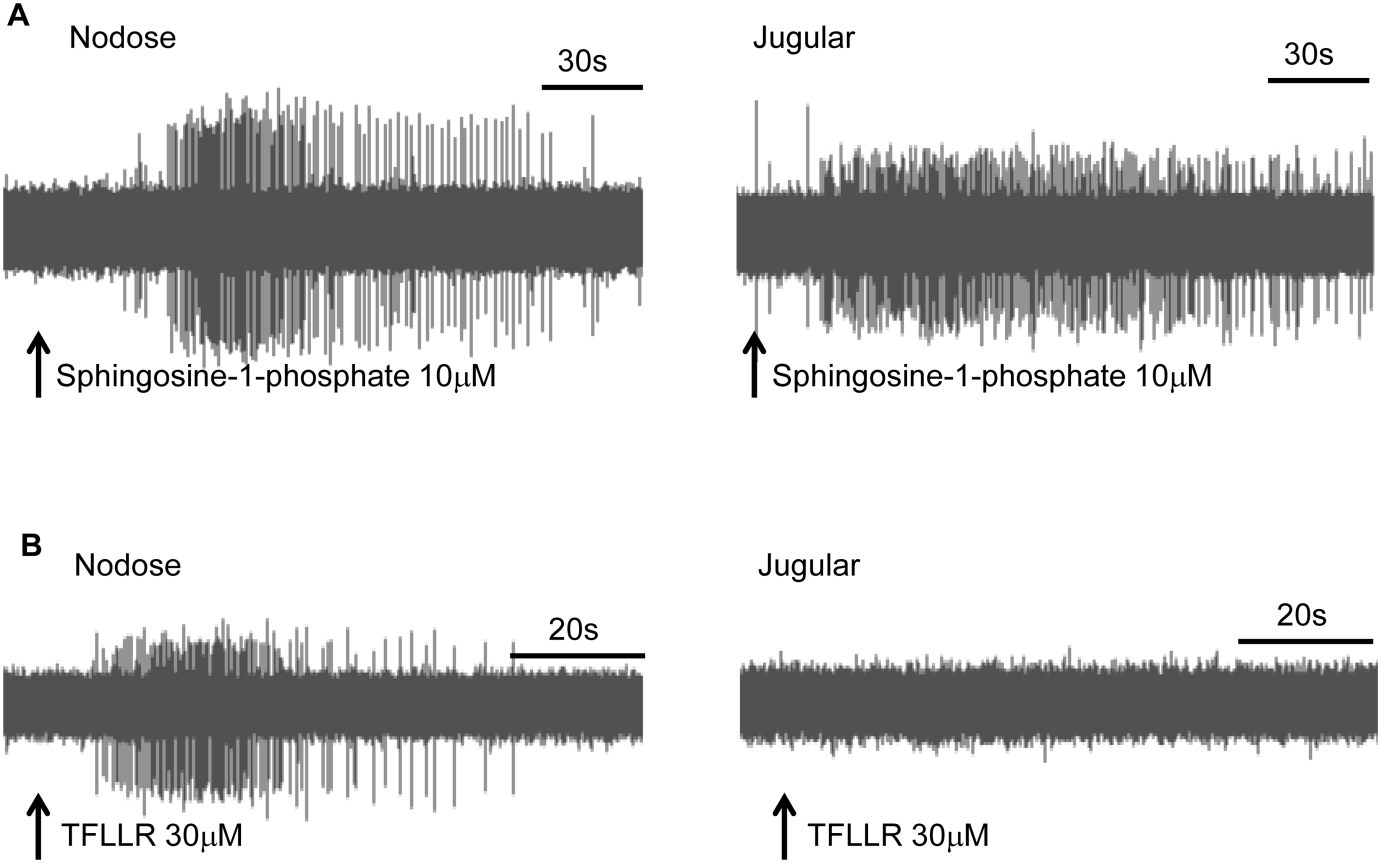
Action potential discharge of a C-fibers terminating in the mouse lungs. (A) Left, representative tracing showing action potential discharge evoked by S-1P in nodose C-fiber terminating in the mouse lung. The peak frequency of action potential discharge was 6±2Hz (N = 10). Right: S-1P evoked action potential discharge in 3 of 5 jugular C-fibers. (B), The PAR1 receptor selective agonist TFLLR stimulated nodose C-fibers (19 ± 3 Hz), but not jugular C-fibers (n = 4).

Stimulation of GPCRs that increase cellular calcium in neuronal cell bodies often lead to action potential discharge at the C-fiber terminals. We determined whether S-1P could stimulate action potential discharge in nodose C-fibers terminating in the mouse lung (Fig 6). In 10 experiments we noted that S-1P (10 μM) stimulated strong action potential discharge in all nodose C-fibers averaging 6 ± 2 Hz. In three experiments the extrapolated EC50 for S-1P averaged 1.1 ± 0.4 μM. For comparison purposes capsaicin (1 μM) stimulated the C-fibers with an average of 30 ± 5 Hz.

We previously noted that thrombin and the PAR1 selective agonist TFLLR activated the majority of vagal C-fibers in the mouse lung, and this was absent in PAR1 -/- mice [31]. In this study we addressed the hypothesis, based on the RNAseq data above, that the PAR1 activation will be selective for nodose C-fibers. TFLLR (3 μM) strongly stimulated 20 of 24 C-fibers with a peak frequency of discharge of 19 ± 3 Hz (Fig 6). The four C-fibers that were unresponsive to TRFLLR were also unresponsive to the α,β-methylene-ATP and we have previously reported those C-fiber terminals in the lungs that lack a response to this P2X_3_ agonist are derived from jugular neurons [12].

## Discussion

We used two methods to characterize the global gene expression in capsaicin-sensitive jugular and nodose C-fiber neurons using ultra-low input RNAseq technology. In the first method, the response to capsaicin was evaluated in a calcium assay, and thus every neuron subjected to RNAseq analysis was by definition capsaicin-sensitive. The disadvantage is that the physiological study may have led to the up- or down-regulation of some genes. In the second method, we used *Trpv1-*Td-tomato reporter mice. This reporter was validated by the observation of *Trpv1* expression only in the Trpv1-tdTomato-positive neurons (Fig 4). This method is less laborious and the isolated neurons are not subjected to physiological investigation, but has the potential disadvantage in that some neurons may have expressed *Trpv1* only during development and are not truly reflective of capsaicin-sensitive C-fiber neurons in the adult animals [47]. Given the potential differences in the two methods, we still saw satisfying consistency between the two RNAseq datasets. Based on functional evaluation of single nodose and jugular C- fibers in the lungs and esophagus, along with single cell RT-PCR, and/or immunohistochemical analysis, several consistent distinctions have been discovered in nodose vs. jugular C-fibers in mice and guinea pigs with respect to expression of ionotropic receptors, GPCRs, neurotrophic factor receptors, and neuropeptide content. For all these genes, the predictions of differential gene expression were consistent with the RNAseq data with either method of selection for the *Trpv1* expressing neuron phenotype, confirming the quality of the isolation and RNAseq (Table 1).

The transcriptome profiling revealed a number of genes with significant expression difference between nodose and jugular C-fiber neurons. Further, pathway analysis revealed that a significant proportion of these genes are mostly involved in processes related to neuron function, such as ion channel activity and synaptic transmission, supporting the hypothesis that jugular and nodose C-fibers are functionally different. Of particular note were the several transcriptional regulators that were selectively expressed in nodose or in jugular C-fiber neurons, which were detected in both datasets (Fig 3). *Prdm12* is a transcription regulator expressed preferentially in jugular vagal neurons. *Prdm12* regulates gene expression by methylating histone H3 on lysine 9 [48]. Interestingly, a bi-allelic missense mutation of this gene is associated with congenital insensitivity to pain [49]. The transcription factor *Cited1*, *Gm13157* and *Tshz1* were preferentially expressed in jugular C-fiber neurons. Previously it has been reported that Cited1 interacts with Tox3 in the brain, and protects neuronal cells from cell death through induction of anti-apoptotic transcripts and repression of pro-apoptotic transcripts [50]. This indicates that Cited1 may have a role in survival of jugular neurons. Notably, *Tox3* also show significant upregulation in jugular neurons in both datasets, although did not reach the cutoff of CPM>10 in the Trpv1-tdTomato selection dataset. On the other hand, *Nhlh2*, *Phox2b*, *Tbx3* and *Hoxb5* were preferentially expressed in nodose C-fiber neurons. *Nhlh2* has been shown to be an oncogene for neuroblastoma [51–53]. Interestingly, *Hoxb5* showed dominant expression in long-term hematopoietic stem cells in the bone marrow [54], and *Tbx3* has been shown to be essential in the maintenance of mouse embryonic stem cell self-renewal [55]. Previous study in adult rat has reported that massive neurogenesis occurs in nodose ganglia following capsaicin-induced neuronal destruction [56]. Therefore, it will be very interesting to explore the neurogenesis activity in nodose neurons and the role of *Hoxb5* and *Tbx3* in this process.

It has long been appreciated that capsaicin-sensitive C-fibers in both the somatosensory and visceral sensory system can be directly stimulated by inflammatory mediators. However, our knowledge is incomplete as to which mediators are the most relevant for vagal sensory nerves. As expected both nodose and jugular Trpv1-positive neurons expressed receptors for various lipid mediators (Table 2). Among the prostanoids receptors, the prostacyclin receptor *Ptgir* was particularly strongly expressed. This is consistent with prostacyclin increasing the electrical excitability of nodose neurons, and stimulating nodose C-fibers in vivo [57–59]. PGD_2_ is elevated at sites of mast cells activation that may occur in food allergy, atopic asthma, and eosinophilic esophagitis. Interestingly, the prostaglandin D receptor (*Ptgdr*) was selectively expressed in jugular neurons. Maher et al. noted that this receptor is expressed in guinea pig jugular neurons, and PGD_2_ can stimulate calcium increases in jugular C-fiber neurons *in vitro*, and evoke coughing *in vivo* [60]. The sphingosine 1-P receptor, *S1pr3*, was very highly expressed especially in nodose neurons, and *S1pr3* activation has recently been implicated in the vagal C-fiber involvement of airways hyperreactivity associated with allergic airway inflammation of the mouse [37]. If this is indeed the case it would most likely be due to stimulation of nodose more than jugular C-fibers in the respiratory tract. Adding credence to an important role for S-1P is our observation that this mediator can evoked strong action potential discharge from nodose C-fibers in the lungs. There was relatively little expression of histamine and 5-HT receptors with the exception of 5-HT_3_. We have previously noted that 5-HT strongly stimulated nodose but not jugular C-fibers via the ionotropic 5-HT_3_ receptors, and this is in keeping with the selective expression of 5-HT_3_ seen here in nodose neurons [29, 30].

There were relatively few receptors for chemokines, cytokines, or toll-like receptors expressed by vagal Trpv1-expressing neurons. A notable exception was *Cxcr2* which was found in both nodose and jugular Trpv1-expressing neurons. This is the receptor for several CXCL chemokines commonly produced at sites of inflammation [61, 62]. Since vagal C-fibers often terminate in the mucosal layer where CXCL8 is produced, the role of this receptor in vagal nociceptor activation is deserving of further study. Another interesting observation was the selective expression of the *Il4ra* in jugular C-fiber neurons, as Il4 is a cytokine elevated in Th2 driven visceral inflammatory disorders. Both nodose and jugular Trpv1-expressing neurons expressed multiple receptors for interferons. There has been little attention given to role vagal afferent nerves in disorders where interferons are elevated, although in vitro studies in DRG neurons support the hypothesis of a autocrine role for interferons in sensory nerve regulation[63]. It should be pointed out that the results were obtained from neurons isolated from healthy mice. The receptors for inflammatory mediators may be up- or down-regulated in the face of visceral inflammation.

The mRNA for receptors expressed in C-fiber neurons as detected our RNAseq analysis was found to predict functional receptors at least with respect to Cxcl2, interferon receptors, and S-1P receptors. Stimuli for these receptors caused rapid and robust increases in calcium in a sizeable subset of capsaicin-sensitive neurons, and in the case of S-1P led to strong action potential discharge at vagal C-fiber terminals innervating the lungs. The prediction based on the RNAseq analysis that PAR1 stimuli would selectively stimulate nodose but not jugular C-fibers was also satisfied in our functional analysis of vagal bronchopulmonary C-fibers. We noted very low levels of expression of Il5ra in nodose and jugular neurons. Recently IL-5 has been shown to stimulate mouse vagal sensory neurons suggesting that in some cases even low level expression may lead to functionally relevant quantities of protein [64].

Some qualifiers are in order when interpreting the RNAseq data. It is possible that contaminating cells contributed to the transcriptome analysis. We do not, however, feel this was a major problem in the present study. Assiduous attention was given to picking with the patch pipette only “clean” neurons without obvious adhering cells. Moreover, microglial cells are known to express various cytokine and chemokine receptors that were not noted in our data set. It also cannot be excluded that the harvest and dissociation of the neurons induced changes in expression of some genes for example because of axotomy. A larger concern pertains to potential heterogeneity of vagal sensory neurons. We reduced this heterogeneity by differentiating nodose from jugular neurons, and further by focusing only on the capsaicin-sensitive class. Nevertheless, vagal neurons innervate virtually all visceral tissues, and there is likely to be subtle distinctions in capsaicin-sensitive nodose and jugular nerve phenotypes innervating the lungs, heart, liver, gut etc. The neurons evaluated were isolated randomly so that those innervating the different organs were not equally represented; most likely neurons innervating some organs were absent, whereas other organs may have been over-represented. This can explain why some gene may be largely expressed in one sample but not in another. For this reason a lack of expression of a target revealed here should not be taken as a categorical lack of expression in vagal neurons, but rather as an indication that the target is probably not expressed in the majority of capsaicin-sensitive neurons. To seriously investigate vagal sensory neurons one must first retrogradely label the neurons that specifically innervated the organ tissue in question. The data presented here support the validity of this approach in that meaningful predictable results (Table 1) can be obtained from a number of neurons easily obtained in retrograde labeling studies. With these qualifications in mind, the data presented will be a useful starting point for those interested in vagal nociceptor innervation of the viscera.

## Supporting information

S1 Table RNA sequencing group comparison summary between Trpv1 positive nodose and jugular neurons from Trpv1-tdTomato selection. (XLSX)

S2 Table RNA sequencing group comparison summary between capsaicin responsive nodose and jugular neurons from capsaicin selection. (XLSX)

S3 Table RNA sequencing group comparison summary between Trpv1 positive nodose and Trpv1 negative nodose neurons from Trpv1-tdTomato selection. (XLSX)
